# FTIR Monitoring of Polyurethane Foams Derived from Acid-Liquefied and Base-Liquefied Polyols

**DOI:** 10.3390/polym16152214

**Published:** 2024-08-03

**Authors:** Yuliya Dulyanska, Luísa Cruz-Lopes, Bruno Esteves, Raquel Guiné, Idalina Domingos

**Affiliations:** 1 Centre for Natural Resources, Environment and Society-CERNAS-IPV, Av. Cor. José Maria Vale de Andrade, 3504-510 Viseu, Portugal; ydulyanska@esav.ipv.pt (Y.D.); bruno@estgv.ipv.pt (B.E.); raquelguine@esav.ipv.pt (R.G.); ijd@estgv.ipv.pt (I.D.); 2 Department of Environmental Engineering, Polytechnic University of Viseu, Av. Cor. José Maria Vale de Andrade, 3504-510 Viseu, Portugal; 3 Department of Wood Engineering, Polytechnic University of Viseu, Av. Cor. José Maria Vale de Andrade, 3504-510 Viseu, Portugal; 4 Department of Food Engineering, Polytechnic University of Viseu, Av. Cor. José Maria Vale de Andrade, 3504-510 Viseu, Portugal

**Keywords:** acid liquefaction, base liquefaction, compressive strength, FTIR, polyurethane foams

## Abstract

Polyalcohol liquefaction can be performed by acid or base catalysis, producing polyols with different properties. This study compared the mechanical properties of foams produced using polyols from liquefied *Cytisus scoparius* obtained by acid and base catalysis and using two different foam catalysts. The differences were monitored using FTIR analysis. Acid-catalyzed liquefaction yielded 95.1%, with the resultant polyol having an OH index of 1081 mg KOH/g, while base catalysis yielded 82.5%, with a similar OH index of 1070 mg KOH/g. Generally, compressive strength with dibutyltin dilaurate (DBTDL) ranged from 16 to 31 kPa (acid-liquefied polyol) and 12 to 21 kPa (base-liquefied polyol), while with stannous octoate (TIN), it ranged from 17 to 42 kPa (acid) and 29 to 68 kPa (base). Increasing water content generally decreased the compressive modulus and strength of the foams. Higher water content led to a higher absorption at 1670 cm^−1^ in the FTIR spectrum due to the formation of urea. Higher isocyanate indices generally improved compressive strength, but high amounts led to unreacted isocyanate that could be seen by a higher absorption at 2265 cm^−1^ and 3290 cm^−1^. DBTL was shown to be the best foam catalyst due to higher trimer conversion seen in the spectra by a higher absorption at 1410 cm^−1^. Acid- and base-derived polyols lead to different polyurethane foams with different FTIR spectra, particularly with a higher absorption at 1670 cm^−1^ for foams from acid-derived liquefaction.

## 1. Introduction

Polyurethane foams (PUFs) derived from lignocellulosic materials represent an environmentally friendly and sustainable alternative to traditional petroleum-based polyurethane foams [1]. Lignocellulosic materials, such as wood, agricultural residues, and other plant-based sources, can be converted into liquid form through various processes like liquefaction or hydrothermal treatment. The resulting liquefied lignocellulosic materials can then be used as a renewable feedstock for the synthesis of polyurethane foams. Among the lignocellulosic materials used for the production of polyurethane foams, wood is probably the most used [2,3,4,5,6], and several agro-industrial residues are also used [7]. The use of renewable lignocellulosic materials reduces dependence on fossil fuels, making the process more sustainable. These bio-based polyurethane foams’ carbon footprint is generally lower than traditional petroleum-based foams. Research and development in this field aim to optimize the production process, enhance foam properties, and make the technology economically viable on a larger scale. Using agricultural residues and waste biomass for polyurethane foam production aligns with the circular economy and sustainable development principles.

There are two main procedures for producing polyols from lignocellulosic materials to be used in polyurethane foam production: oxypropylation and liquefaction. Oxypropylation of biomass involves the addition of propylene oxide to biomass-derived compounds containing hydroxyl groups. This process is a way to modify the structure of biomass-derived materials, making them more suitable for various applications, including the production of bio-based polyols for polyurethane foams. It is generally performed with high pressure and temperature using KOH as a catalyst [8]. This process has been used for several lignocellulosic materials such as cork [9,10]. The liquefaction process with polyalcohols offers several advantages, such as promoting the solubility of biomass components and facilitating solvolytic reactions that lead to the breakdown of complex structures. Polyalcohols, which are compounds with multiple hydroxyl (OH) groups, can be used as solvents or reactants in the liquefaction process. Common polyalcohols used in liquefaction include glycerol, ethylene glycol, or polyethylene glycols (PEGs) [11,12,13,14]. This process can have acid [15,16,17] or base catalysis [11,12,18], and the choice of catalyst depends on the initial material. Generally, acid catalysts lead to higher liquefaction yields, but base catalysts are preferred for materials with suberin content, like cork barks [19].

The selection of liquefaction solvents depends on the intended properties of the liquefied material. The most important properties are hydroxyl number, acid number, viscosity, and molecular weight. According to Hu et al. [8], polyols obtained by polyalcohol liquefaction generally exhibit hydroxyl numbers between 100 and 600 mg KOH/g, acid numbers from 0 to 40 mgKOH/g, viscosities from 300 to 4500 cps, and molecular weight (MW) from 250 to over 7000 gmol^−1^. Nevertheless, these parameters depend on the type and amount of solvent used in the liquefaction procedure. For example, glycerol has been reported to have a hydroxyl value of around 1800 mg KOH/g [20], while PEG200, PEG400, and PEG600 have significantly lower hydroxyl values of 534–590, 268–294, and 178–196 mg KOH/g, respectively [21]. Therefore, the amount of polyalcohols used in the liquefaction process influences the final hydroxyl value. For instance, the liquefaction of corn stover with different glycerol/corn stover ratios led to different hydroxyl values, ranging from 267 mg KOH/g to 346 mg KOH/g for 1:2 and 1:5 corn stover/glycerol ratios [22]. The hydroxyl number is important for polyurethane (PU) production and is known to decrease along the liquefaction, which has been considered to be due to the consumption of hydroxyl in oxidation and dehydration reactions [8,23,24]. Another important property, viscosity, usually also decreases with the progress of the liquefaction process [8]. However, D’Souza and Yan [25], who studied the effect of temperature on the production of bark-based polyols through liquefaction, stated that for higher liquefaction temperatures, the polyols exhibited an elevated viscosity, accompanied by an increase of the MW distributions.

In PU production, polyols can have from two to eight reactive hydroxyl (OH) groups present in a polyol molecule and a molecular weight from 200 to 8000 gmol^−1^. Therefore, polyurethane properties can be adjusted according to the needs [8]. The formation of polyurethane foams involves a complex reaction known as polyurethane synthesis, which typically consists of two main reactions: the polyol–isocyanate reaction (foam formation) and the polyol–water reaction (blowing reaction). These reactions occur simultaneously and form a three-dimensional polymer network with the characteristic properties of polyurethane foam. Catalysts facilitate the polymerization reaction between polyols and isocyanates to form the polyurethane matrix. Common catalysts used in the production of polyurethane foams include tertiary amine compounds, such as triethylenediamine (TEDA) or dimethylcyclohexylamine (DMCHA), or organotin compounds, such as stannous octoate (Tin(II) 2-ethylhexanoate, TIN) and dibutyltin dilaurate (DBTDL), and are also widely employed [26]. Tertiary amines catalyze both formation and expansion reactions, but they exhibit a distinctive characteristic wherein they demonstrate significantly higher efficacy in the isocyanate–hydroxyl reaction when employed with aliphatic isocyanates, contrasting with the combination with aromatic isocyanates [26].

Blowing agents, such as water or certain hydrocarbons, are used to generate gas during the reaction, leading to the formation of foam [1]. Water, in particular, reacts with isocyanate to release carbon dioxide, contributing to foam expansion. The choice of blowing agent significantly affects the foam properties. For example, Kurańska et al. [27] studied the effects of blowing agent type on the foaming process, cellular structure, mechanical properties, and changes in thermal conductivity during one year of aging and concluded that carbon dioxide exhibited the highest reactivity. Additionally, foams blown with carbon dioxide displayed a cellular structure characterized by smaller cell sizes compared to those using physical blowing agents. The lowest thermal conductivity was, however, observed in polyurethane systems foamed with isopentane and a mixture of isopentane and cyclopentane.

Silicone surfactants are often added to improve the cell structure and overall foam properties. They help in controlling the size and distribution of cells in the foam. Zhang et al. [28] studied the role of silicone surfactant in flexible polyurethane foams using different siloxane-to-polyether ratios and concluded that silicone surfactants with a higher silicone content had lower surface tension, resulting in smaller bubble size and an increased bubble generation rate but leading to unstable foams. On the other hand, surfactants with a siloxane-backbone-to-polyether ratio ranging from 0.32 to 0.5 demonstrated a balanced performance, exhibiting a moderate equilibrium between surface tension and lamella elasticity. Lower surface tension generally leads to lower cell sizes and higher closed-cell content [29]. These authors also stated that the reduction in cell size also leads to lower thermal conductivity of foams, showing a linear relationship between the two variables across a broad range of cell sizes [29]. Similar results were presented for rigid polyurethane foams, where a smaller cell size enhanced the thermal insulation properties of rigid PUFs and was considered to be a crucial factor in decreasing the thermal conductivity of the foams [30].

Acid or base liquefaction yield depends on the type of the lignocellulosic material. Generally, alkaline hydrolysis is better for barks with higher cork content since an alkaline pH is needed for the saponification of suberin, which is the main content of cork. This was proven before, for example, by Yona et al. [19] for the liquefaction of *Quercus suber* bark that yielded around 61–85% for alkaline catalysis and 43–50% for acid catalysis or for Douglas fir bark, which also has a high suberin content and yielded around 80% for alkaline hydrolysis and 30% for acid hydrolysis [11]. For lignocellulosic materials without suberin acid, catalysis is more efficient [18].

Polyurethanes foams are synthesized by reacting polyols with isocyanates, which are both derived from non-renewable sources [31]. In our study, we used polyols produced from biomass, specifically wood, to synthesize a sustainable, biodegradable class of polyurethanes. The subsequent step involves developing sustainable, eco-friendly strategies that minimize impacts on human health and the environment. To reduce the consumption of 4,4′-methylene diphenyl diisocyanate (MDI), as proposed by Domingos et al. [6], we precisely determined the hydroxyl (OH) groups in the polyol and calculated the exact amount of isocyanate needed for polyurethane production, thereby mitigating the health and environmental impacts of using excess isocyanate.

Additionally, some researchers are exploring strategies to synthesize non-isocyanate-based polyurethanes or propose using isocyanates derived from renewable sources [32]. Others suggest using plant-based sources to obtain dicarboxylic acids, which can be subsequently transformed into diisocyanates using conventional solvents such as tetrahydrofuran (THF). As an example, 5-methyl-THF, a “greener” solvent, can be used in the synthesis of diisocyanates [33]. An alternative approach, proposed by Smith et al. [34], involves the oxidation of polyol fractions to polycarboxylic acids, which are then converted into polyisocyanates that react with remaining polyol fractions, resulting in polyurethanes derived entirely from renewable sources.

In this study, a comparison was made between the mechanical properties of the foams with varying percentages of two different catalyzers, blowing agents, and isocyanate for both acid- and base-catalyzed liquefaction. The changes were monitored by FTIR.

## 2. Materials and Methods

The reagents used for the preparation of polyol included glycerol (Sigma-Aldrich, St. Louis, MI, USA, ≥99.5%), ethylene glycol (≥99.8%, St. Louis, MI, USA, Sigma-Aldrich, USA), sulfuric acid (analytical grade, Sigma-Aldrich, St. Louis, MO, USA), methanol (A.C.S. grade, ≥99.8%, Sigma-Aldrich, St. Louis, MO, USA), sodium hydroxide (analytical grade, Sigma-Aldrich, St. Louis, MO, USA), ethanol (analytical grade, absolute, Fisher Chemical, Hampton, NH, USA, >99.8%), and ultrapure water (analytical grade, Sigma-Aldrich, St. Louis, MO, USA). For foam preparation, as catalyzer, di-n-butyltin dilaurate (95%, Sigma-Aldrich, St. Louis, MO, USA) and tin(II) 2-ethylhexanoate (95%, Sigma-Aldrich, St. Louis, MO, USA); as surfactant, Tegostab B8404^®^ (Evonik Industries, Essen, Germany); isocyanate MDI Voranate M229^®^ (Dow Chemical Company, Midland, MI, USA), with an average functionality of 2.7 and a %NCO of 31.1%; and ultrapure water were utilized.

### 2.1. Sample Liquefaction

The *Cytisus scoparius* samples underwent a drying process in an oven at 100 °C and were finely ground to enhance their surface area. A precisely measured 10 g sample was then weighed and transferred into the reactor. Subsequently, a mixture of glycerol and ethylene glycol in a 50:50 ratio, along with 3% sulfuric acid based on the sample weight (used as a catalyst), was introduced. The glycerol–ethylene glycol mixture was added to fully cover the wood sample, and the Parr reactor was tightly sealed to prevent any potential leaks. The agitator was turned on at 75 rpm to ensure a complete mixture of the sample with the solvents. The temperature was gradually increased to 180 °C and maintained at this level for 60 min. After the designated time, the reactor was cooled to room temperature. Upon opening the reactor, the liquefied wood product was collected. This product, dissolved in 100 mL of methanol, underwent filtration for further processing. Samples of *Cytisus scoparius* were also liquefied with alkaline catalysis. In this case, potassium hydroxide (KOH) was used as the catalyst, with a maximum of around 6%.

Sulfuric acid was used to catalyze the synthesis of polyol under acidic conditions from glycerol, ethylene glycol, and lignocellulosic material. There is no known chemical reaction that can describe all processes in polyol synthesis, since reactions differ between different hemicelluloses, cellulose, or lignin. In the synthesis of polyol, sulfuric acid acts as a catalyst facilitating the reaction between glycerol, ethylene glycol, and lignocellulosic material; therefore, it is not consumed in the reaction. The presence of sulfuric acid helps in the esterification and etherification reactions, leading to the formation of polyol compounds. Specifically, sulfuric acid aids in breaking down and modifying the complex lignocellulosic structure, enabling the incorporation of glycerol and ethylene glycol into the polyol matrix. This acidic environment promotes the desired chemical transformations that are essential for obtaining the polyol with the desired properties for further applications, such as in foam production or other industrial processes.

Cellulose is mainly hydrolyzed into glucose [23], while hemicelluloses are hydrolyzed mainly into its monomeric sugars. Lignin undergoes depolymerization and solubilization in the presence of the acidic medium and polyhydric alcohols [13,35]. First, depolymerization of lignin into smaller phenolic fragments occurs, followed by the esterification of these fragments with glycerol and ethylene glycol to enhance solubility. These reactions collectively break-down the wood’s lignocellulosic structure, resulting in a liquefied mixture of various organic compounds.

### 2.2. Determination of Hydroxyl Value

The OH index was determined through potentiometric titration of the residual acetic acid present after the esterification of free OH groups. An approximate weight of 20 mg of the sample was placed in a screw-cap tube. Subsequently, 0.1 mL of acetylation mixture was added, which had been prepared just prior to the analysis by mixing 4.7 mL of acetic anhydride with 4 mL of pyridine. The tube’s content was then homogenized and kept for 24 h in an oven set at 50 °C. Following cooling to room temperature, the mixture was transferred quantitatively to a 100 mL beaker using 10 mL of acetone, and an equal amount of distilled water was added. The mixture was then titrated using a potentiometric method with standardized 0.1 N LiOH. The average value of three replicates was obtained, and the number of milligrams of KOH required to neutralize one gram of the sample was calculated using the following equations:

(1) $$OH(\%)=\frac{\left[\left(ms\times \frac{Vb}{mb}\right)-V\right]\times f\times 1.7\times 100}{W}$$

(2) IOH(mg KOH/g)=33×OH(%)

where V is the volume of LiOH solution required for the titration of the sample (mL); Vb is the volume of LiOH solution required for the titration of the blank (mL); ms is the acetylating mixture of the sample (mg); mb is the blank (acetic anhydride and pyridine) in mg; f is the standardized titer of LiOH solution; W is the weight of the sample (mg); 1.7 is the mass, in mg, of OH groups equivalent to 1 mL of 0.1 M LiOH.

### 2.3. Foam Preparation

Approximately 4 g of neutralized and dried polyol was weighed and placed in a polypropylene container on a stable surface. The measured isocyanate was added to the polyol in a cylindrical container with a dimension of 60 mm × 120 mm (diameter × height) using a syringe. The surfactant was introduced into the mixture to stabilize the foam by controlling the size and distribution of bubbles. The measured amount of water was then added as the blowing agent, reacting with isocyanate to release carbon dioxide, contributing to the expansion of the foam. The mixture underwent mixing at 2000 rpm for 1 or 2 min, ensuring homogeneity and initiating reactions effectively. Subsequently, the catalyst (DBTDL or TIN) was added to the mixture to facilitate and accelerate the reaction between polyol and isocyanate, promoting the foaming process. Further mixing at 2000 rpm for 1 or 2 min followed until the foam started to rise. It was allowed to rise freely at room conditions. Generally, 4 g of polyol, 0.4 g of water (10%), 0.28 g of surfactant (7%), 0.2 g of catalyzer (5%), and 11 g isocyanate were used. Variations were made for water (0.2 g to 0.8 g), catalyzer (0.1 g to 0.4 g), and isocyanate 9–13 g

### 2.4. Foam Testing

The polyurethane foam sample was prepared by cutting it into a cylinder with approximately 60 mm diameter and 30 mm height. The polyurethane foam sample was placed between the compression plates. The testing parameters, including compression speed and limit, were adjusted. The compression speed was 5 mm/min. The Universal Test Machine was started, applying a gradual and uniform compression force to the polyurethane foam.

During the test, data on applied force and corresponding deformation were recorded in real time using testing software. The compression continued until the sample underwent deformation and the force stabilized, indicating a significant portion of the foam had compressed.

### 2.5. FTIR Analysis

Foams were dried overnight in an oven at 102 ± 2 °C and ground in a mortar. A Perkin Elmer UATR Two FT-IR Spectrometer (Beaconsfield, UK) was used with a resolution of 4.0 cm^−1^, recording 72 scans in the range of 4000–400 cm^−1^. The powder was placed directly on the crystal, completely covering the surface. Three spectra were collected for each sample.

## 3. Results and Discussion

### 3.1. Mechanical Properties

Acid catalysis led to a 95.1% yield, and the resultant polyol presented an OH index of 1081 mg KOH/g and an acid number of 2.56 mg KOH/g (ethanol blank), while base catalysis had an 82.5% yield and an OH index of 1070 mg KOH/g and an acid number of 2.85 mg KOH/g (ethanol blank). Therefore, no significant difference has been observed for base- or acid-liquefied polyols, although acid liquefaction was more efficient, leading to a higher amount of polyol. The high hydroxyl value of the polyols is due to the high amount of solvent used in the liquefaction procedure with 1:10 ratio sample:solvent. The solvent used was glycerol/ethylene glycol (50:50), and both have high OH index, ethylene glycol (IOH = 1808 mg KOH/g), and glycerol (IOH = 1827 mg KOH/g) in accordance with Chajęcka [36].

**Figure 1:**
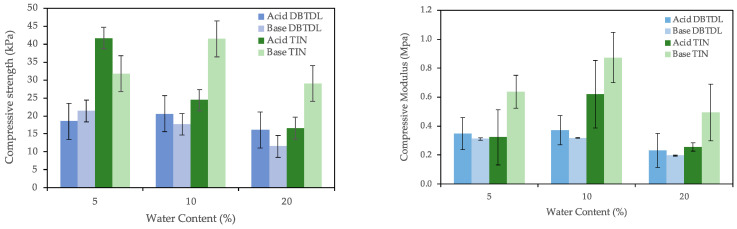
Compressive strength and compressive modulus of PUF made with different amounts of blowing agent (water). It was prepared using as a base 4 g of polyol, 11 g of isocyanate, 0.2 g of catalyst (TIN and DBTDL), 0.28 g of surfactant, and the blowing agent varied from 0.2 to 0.8 g.

Water was chosen as the blowing agent. Figure 1 shows the variation of compressive modulus and compressive strength for increasing water content using DBTDL and TIN catalyzers. Overall, under the same conditions, PUFs made with a TIN catalyzer presented higher compressive strength and compressive modulus.

The compressive strength of the foams using DBTDL catalyzer varied between 16 kPa and 31 kPa for the acid-liquefied polyol and 12–21 kPa for the base-liquefied polyol. In relation to the foams made with TIN catalyst, compressive strength varied between 17 kPa and 42 kPa and 29 kPa and 32 kPa for base-liquefied polyol. Therefore, results show that the compressive strength of foams with TIN catalyst is higher than the ones using DBTDL. Nevertheless, all of them are smaller than the compressive strengths of 80–150 kPa obtained for PU foams produced with polyols with high biomass content (ca. 50%) produced by the combined liquefaction of wood and starch by Yao et al. [37] or PUF with polyols obtained by liquefaction of several waste papers (68 to 195 kPa) by Hu et al. [8]. The maximum compressive strength of about 21 kPa (DBTDL) and 42 kPA were obtained for 5% water content. The compressive strength reported by Li et al. [38] for 7% water content was 147 kPa; however, the polyether polyol used was prepared from a mixture of sugar and glycerol catalyzed with potassium hydroxide at low temperature (90 °C).

**Figure 2:**
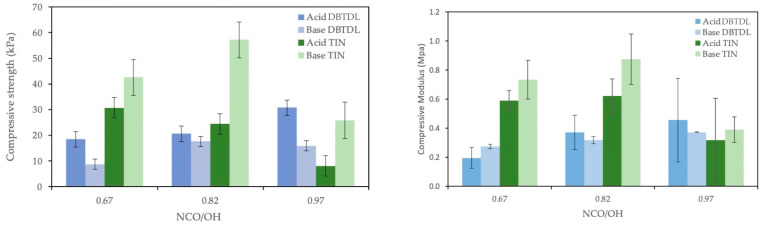
Compressive strength and compressive modulus of PUF made with different isocyanate indices. It was prepared using 4 g of polyol, 0.4 g of various blowing agents, 0.2 g of catalyst (TIN and DBTDL), 0.28 g of surfactant, and isocyanate ranging from 9 to 13 g.

**Figure 3:**
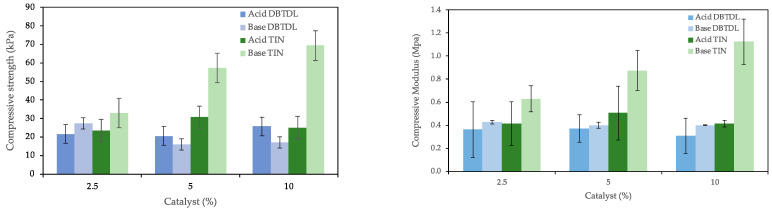
Compressive strength and compressive modulus of PUF made with different amounts of catalyst (DBTDL) for TIN catalyst. It was prepared using as a base 4 g of polyol, 0.4 g of various blowing agents, 0.28 g of surfactant, 11 g of isocyanate, and the catalyst varied from 0.1 to 0.8 g for TIN and DBTDL.

Compressive modulus seems to follow a similar trend for foams using DBTDL catalyst, varying between 233 kPa and 371 kPa for acid-liquefied polyol and between 197 kPa and 319 kPa for the base-liquefied one. These values are also lower than the ones reported before, for example, by Lee et al. [39], who reported 800 to 3400 kPa for a biodegradable polyurethane foam produced from liquefied waste paper. Nevertheless, mechanical properties depend on the equilibrium between the blowing agent, catalyzer, and isocyanate, and higher mechanical properties can be achieved, as reported in Figure 2 and Figure 3.

Results show that with the increase in water content, there was a decrease in both compressive modulus and compressive strength for the foams using DBTDL catalyzer. This decrease was observed for both acid- and base-liquefaction-derived polyols. The results using TIN catalyzer are less consistent with high standard deviation but generally have a similar trend, although the highest values are obtained for 10% water content. Similar results were presented before for other lignocellulosic polyols, for instance, for polyurethane foams from liquefied *Eucalyptus globulus* branches [6] or orange peel waste [40] using tertiary amines catalyzers. The decrease can be associated with the higher expansion of the foam, producing a less densified structure and, therefore, a more resistant matrix. The same was observed before, for example, by Li et al. [38], who studied the influence of the water content on the apparent density and compressive strength of rigid foams reaching a compressive strength from 147 kPa (7% water content) and 401 kPa (3% water content) or Hakim et al. [41], who reported the compressive strength variation of rigid polyurethane foam prepared from sugarcane bagasse polyol.

Figure 2 presents the variation of compressive strength and compressive modulus for different NCO/OH ratios (isocyanate index). Compressive strength for the index value of 0.67 was around 18 kPa for acid-liquefaction-derived polyol and under 10 kPa for base-liquefied polyol using DBTDL as the catalyst. With the increase in NCO/OH ratio, compressive strength increased, reaching a maximum of 31 kPa for acid-liquefied polyol. In relation to base-liquefied polyol, there was an increase from 0.67 to 0.82 in the isocyanate index, followed by a decrease for the index value of 0.97. This decrease for the index value of 0.97 may be due to unreacted materials. Higher isocyanate content usually leads to higher mechanical properties, which have been attributed to the increased hard segment content and crosslinking densities in polymer networks [8,42]. Nevertheless, if the amount of isocyanate is too high, the mechanical properties are affected due to incomplete curing of the isocyanate, which has been reported before [8,42]. This is confirmed by the FTIR analysis presented in Section 3.2.

Using TIN catalyst, there was an increase followed by a decrease in both acid-liquefied and base-liquefied polyols’ compressive strength. The highest compressive strength was attained for base-liquefied polyol using TIN catalyst; nevertheless, this is probably due to the lower growth of these foams. Generally, compressive modulus followed a similar trend. This means that with TIN catalyst, an isocyanate index higher than 0.82 is detrimental to the mechanical properties of the foams. Similar results were reported by Hakim et al. [41], who attributed this behavior to an increased blowing effect by the CO_2_ created in extra condensation reactions between the isocyanate groups.

Figure 3 presents the variation in mechanical properties with varying amounts of catalyzers. Higher amounts of catalyst appear to increase compressive strength, which is particularly noticeable with a TIN catalyst. This effect could be caused by the rapid gelling reaction outpacing the expansion reaction. The faster gelling process might hinder the generation of sufficient CO_2_ to facilitate foam expansion. Additionally, the substantial rise in temperature induced by higher catalyst concentrations could enhance the reaction between the hydrogen-bonded with the nitrogen atom in the urethane group and the additional isocyanate, leading to the formation of allophanate [43]. Using DBTDL catalyst, there is no significant difference between acid- and base-derived polyols, but for TIN catalyst, there is a significantly higher compressive strength for base-derived polyol. The highest compressive strength of around 69 kPa was achieved for base-derived polyol using 10% TIN catalyst. Compressive modulus also increases for higher amounts of catalyst. The highest compressive modulus was also achieved for TIN catalyst using base-derived polyol with 1100 kPa.

### 3.2. FTIR Analysis

**Figure 4:**
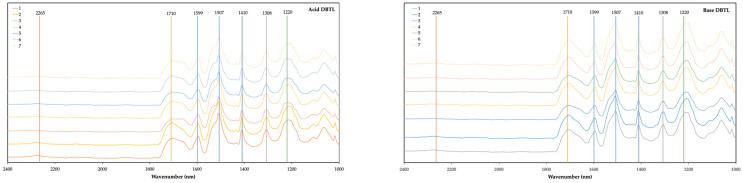
FTIR spectra of foams using acid-liquefied polyol (**left**) and base-liquefied polyol (**right**) and DBTDL as catalyzer. (1) Base foam with 10% water, surfactant (7%), catalyzer (5%), and NCO index 0.8; (2) water 5%; (3) water 15%; (4) 2.5% catalyzer; (5) 10% catalyzer; (6) NCO index 0.7; (7) NCO index 1.

**Figure 5:**
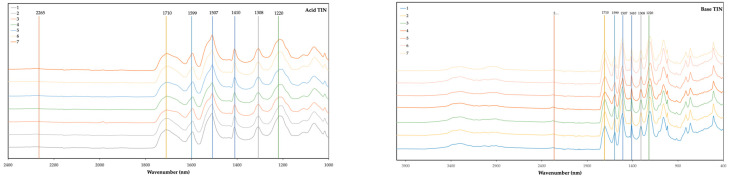
FTIR spectra of foams using acid-liquefied polyol (**left**) and base-liquefied polyol (**right**) and TIN as catalyzer. (1) Base foam with 10% water, surfactant (7%), catalyzer (5%.) and NCO index 0.8; (2) water 5%; (3) water 15%; (4) 2.5% catalyzer; (5) 10% catalyzer; (6) NCO index 0.7; (7) NCO index 1.

Figure 4 and Figure 5 present the FTIR spectra of foams made from acid- and base-liquefied polyols and two different catalysts. For visualization purposes, the spectra are only presented in the 2400–1000 cm^−1^ range. The full spectra can be found in the Supplementary Materials. The main peak assignments are presented in Table 1.

Overall, all the spectra presented the usual peaks of polyurethane foams.

All the foams exhibit a peak at 3290 cm^−1^, which has been assigned to the N-H stretching vibration of urethane groups. No significant differences are observed in this peak at this wavelength. The peaks of unreacted OH and unreacted NCO groups can be found at around 3400 cm^−1^ and 2265 cm^−1^ [44]. Some of the foams still have the peak at 2265 cm^−1^ in conjunction with a higher absorption rate at 3400 cm^−1^, which indicates that there were some unreacted NCO and OH groups. When comparing the foams with higher isocyanate content (NCO index 1 -nº 7) with foams with lower isocyanate content (NCO index 0.7 -nº 6), a higher absorption is visible. Increasing isocyanate content above this point would result in a higher amount of unreacted materials. This might be the reason for the decrease in mechanical strength observed for base-liquefied polyol catalyzed by DBTL and for both acid- and base-derived polyols catalyzed with TIN.

The carbonyl peak has its maximum absorbance at 1710 cm^−1^, which is associated with hydrogen-bonded urethane carbonyl, while free urethane absorbs at 1730 cm^−1^ [45,46,47]. The shoulder at 1670 cm^−1^ is due to urea carbonyl [48]. The foams with higher water content (15% water-nº 3) exhibit a higher urea carbonyl peak, except for base TIN, which has been stated to be due to the formation of more urea as a result of the reaction of isocyanate with water [45]. Similarly, the foams with less water content have a lower urea carbonyl peak (5% water- nº 2). Generally, the foams using the acid-liquefied polyols have a higher absorption at 1670 cm^−1^ compared to the absorption at 1710 cm^−1,^ and this is observed for both catalysts.

The 1593 cm^−1^ band corresponds to the C-C stretching of the aromatic ring which is present in MDI [49]. No significant difference has been found in this peak for both polyols and for different catalysts. The band at around 1500 cm^−1^, with a maximum at 1507 cm^−1^, is generally attributed to bending vibrations of polyurethane N-H groups [50] but can also have some collaboration with C-N stretching. The main difference in this band is observed between acid- and base-derived polyols since the shoulder at around 1530 cm^−1^ seems to be higher for base-liquefied polyols.

The peak at 1410 cm^−1^ is associated with the isocyanurate C-N stretching [51,52]. This peak demonstrates the catalysts’ potency to achieve high trimer conversion, which is crucial for enhancing fire retardancy in rigid foams, as stated before [51]. Overall, this peak is slightly higher for DBTL-catalyzed foams, which might indicate that this catalyst is better when compared to TIN with respect to fire retardancy. Uretidinedione also presents a similar band; nevertheless, it is accompanied by a band in the region 1700–1800 cm^−1^, which is not present in this case [52].

The peak at 1308 cm^−1^ has been assigned to aliphatic C-H bending vibrations [53], while the band around 1220 cm^−1^ can have several contributions. In this case, the maximum is around 1200 cm^−1^.

## 4. Conclusions

In a novel approach, *Cytisus scoparius* was liquefied using either acid or base catalysis to produce polyols with diverse properties. Notably, while acid-catalyzed liquefaction achieved a higher liquefaction percentage, the OH index remained consistent across both types of polyols. Polyurethane foams synthesized with a DBTDL catalyst exhibited similar compressive strength and modulus regardless of the polyol’s origin. However, using a TIN catalyst, the mechanical properties were enhanced in foams derived from base-liquefied polyols. An increase in water content typically reduced the compressive modulus and strength of the foams, correlating with increased absorption at 1670 cm^−1^ due to urea formation. Higher isocyanate indices generally improved compressive strength, although excessive isocyanate led to unreacted residues, as indicated by elevated absorption at 2265 cm^−1^ and 3290 cm^−1^. Absorption at 1410 cm^−1^ identified DBTL as the best catalyst for foam production, attributed to higher trimer conversion, which enhances fire retardancy in polyurethane foams. Distinct FTIR spectra of the polyurethane foams highlighted that those derived from acid-catalyzed liquefaction exhibited higher absorption at 1670 cm^−1^, showing the different structural properties imparted by the catalytic process. The properties of polyurethane foams can be adapted to satisfy specific requirements, which makes them suitable for many applications across different sectors. These foams can be used in packaging and material conditioning. Typical values of compressive strength for low-density packaging rigid foams are in the range 20 to 100 kPa, which is a similar range to our foams [54]. These foams are often used for lightweight and delicate items.

## Figures and Tables

**Table 1:** FTIR spectra assignment of bands in PU Foams.

Wavenumber (cm^−1^)	Peak Assignment
3400	O-H stretching
3290	N-H stretching vibration of urethane groups
2265	Antisymmetric stretching vibration of NCO
1730	C=O stretching (free urethane)
1710	C=O stretching (hydrogen-bonded urethane)
1670	C=O stretching (urea)
1593	C-C stretching of the aromatic ring
1530	C-N stretching of urethane group
1507	N-H bending vibration
1410	C-N stretching of the aromatic ring
1308	Aliphatic C-H bending vibrations
1200–1230	C-N stretching of urethane group
1096	C-O-C stretching

## Data Availability

Data are available on request from the corresponding author. The data are not publicly available due to data are contained within the article.

## Supplementary Materials

The following supporting information can be downloaded at: https://www.mdpi.com/article/10.3390/polym16152214/s1, Figure S1: FTIR spectra of foams using acid-liquefied polyol and DBTDL as catalyzer; Figure S2: FTIR spectra of foams using base-liquefied polyol and DBTDL as catalyzer; Figure S3: FTIR spectra of foams using acid-liquefied polyol and TIN as catalyzer; Figure S4: FTIR spectra of foams using base-liquefied polyol and TIN as catalyzer.
