# Supplementary material for: FTIR Monitoring of Polyurethane Foams Derived from Acid-Liquefied and Base-Liquefied Polyols
Source: Polymers (Basel). 2024 Aug 3;16(15):2214. doi: 10.3390/polym16152214 (PMC11314664; doi:10.3390/polym16152214)
Supplement: Supplementary file 1 [file polymers-16-02214-s001.zip › polymers-3060163-supplementary.pdf]

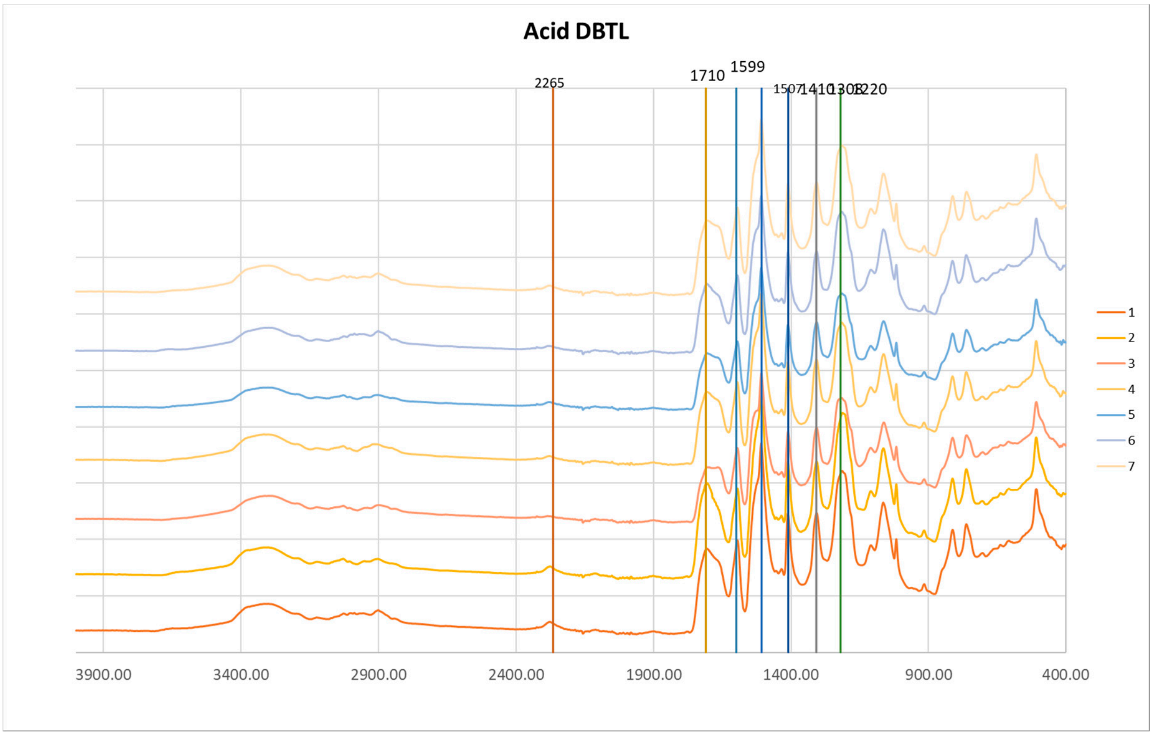

Figure S1: FTIR spectra of foams using acid liquefied polyol and DBTDL as catalyzer;

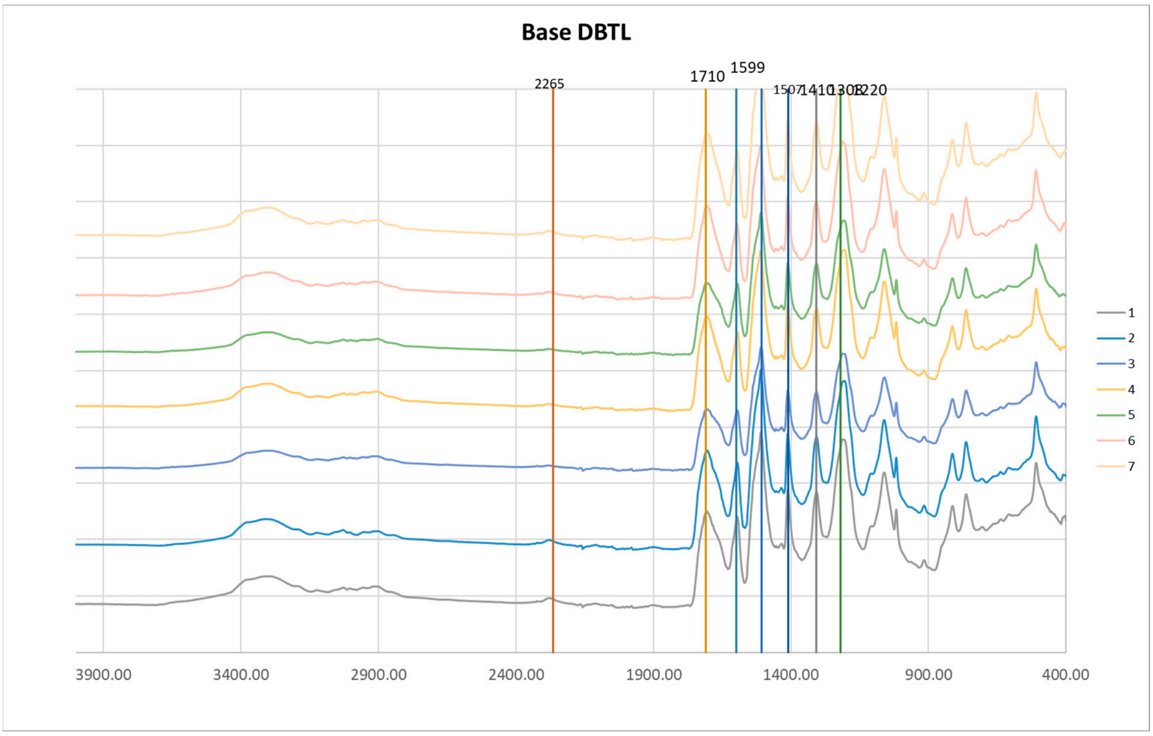

Figure S2: FTIR spectra of foams using base liquefied polyol and DBTDL as catalyzer;

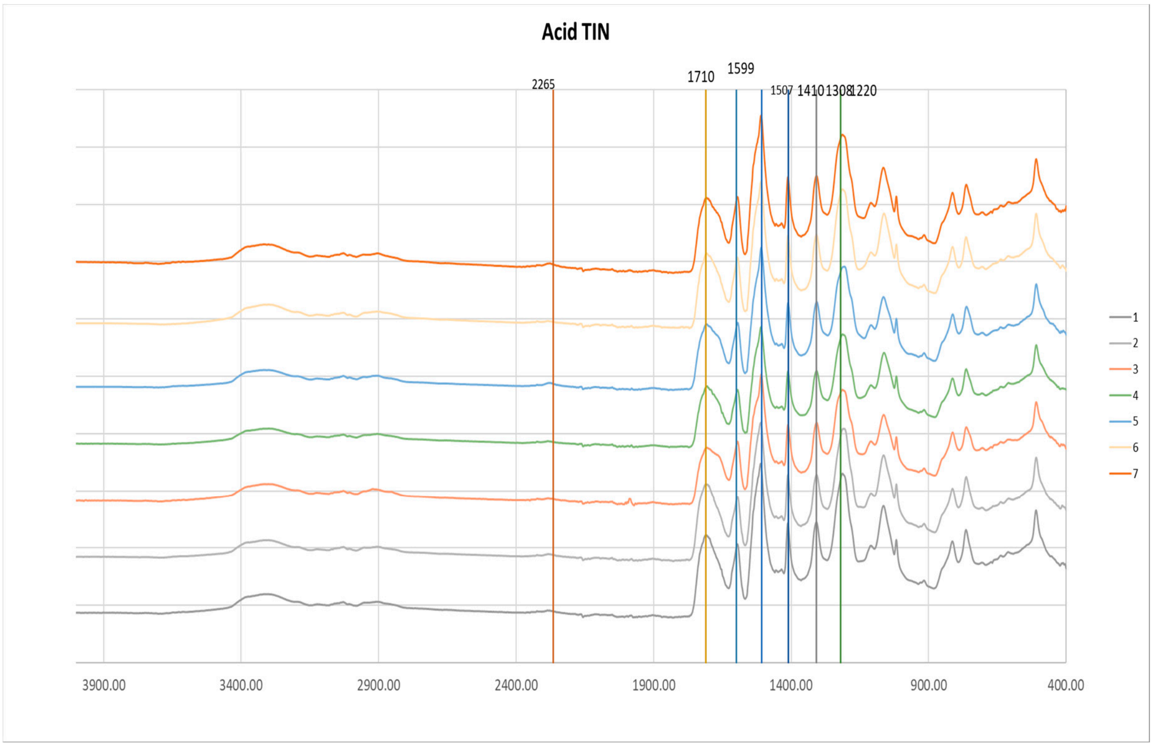

Figure S3: FTIR spectra of foams using acid liquefied polyol and TIN as catalyzer;

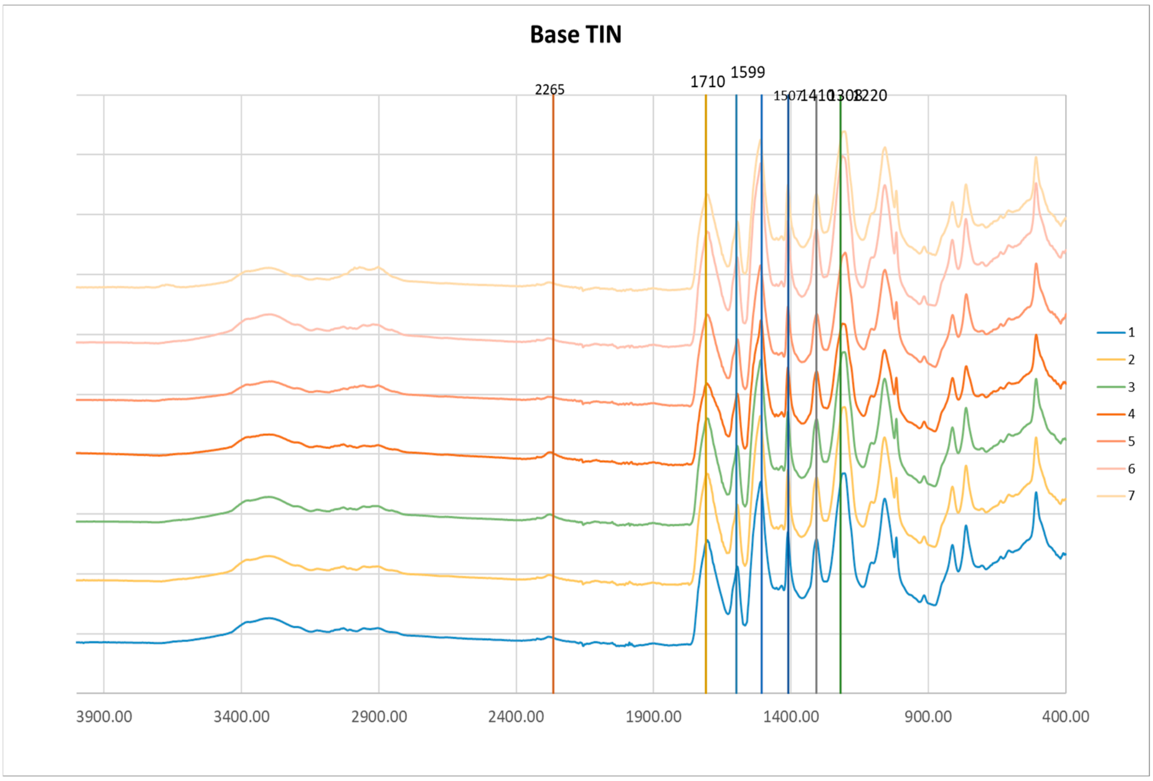

Figure S4: FTIR spectra of foams using base liquefied polyol and TIN as catalyzer;
